# Supplementary material for: The interactome of intact mitochondria by cross-linking mass spectrometry provides evidence for coexisting respiratory supercomplexes
Source: Mol Cell Proteomics. 2017 Dec 8;17(2):216–32. doi: 10.1074/mcp.RA117.000470 (PMC5795388; doi:10.1074/mcp.RA117.000470)
Supplement: Supplemental Data [file supp_RA117.000470_133922_0_supp_23978_fzffwf.zip › spectra_annotation/mito_DR_spectra_annotation/417-1-4-1-15-1.pdf]

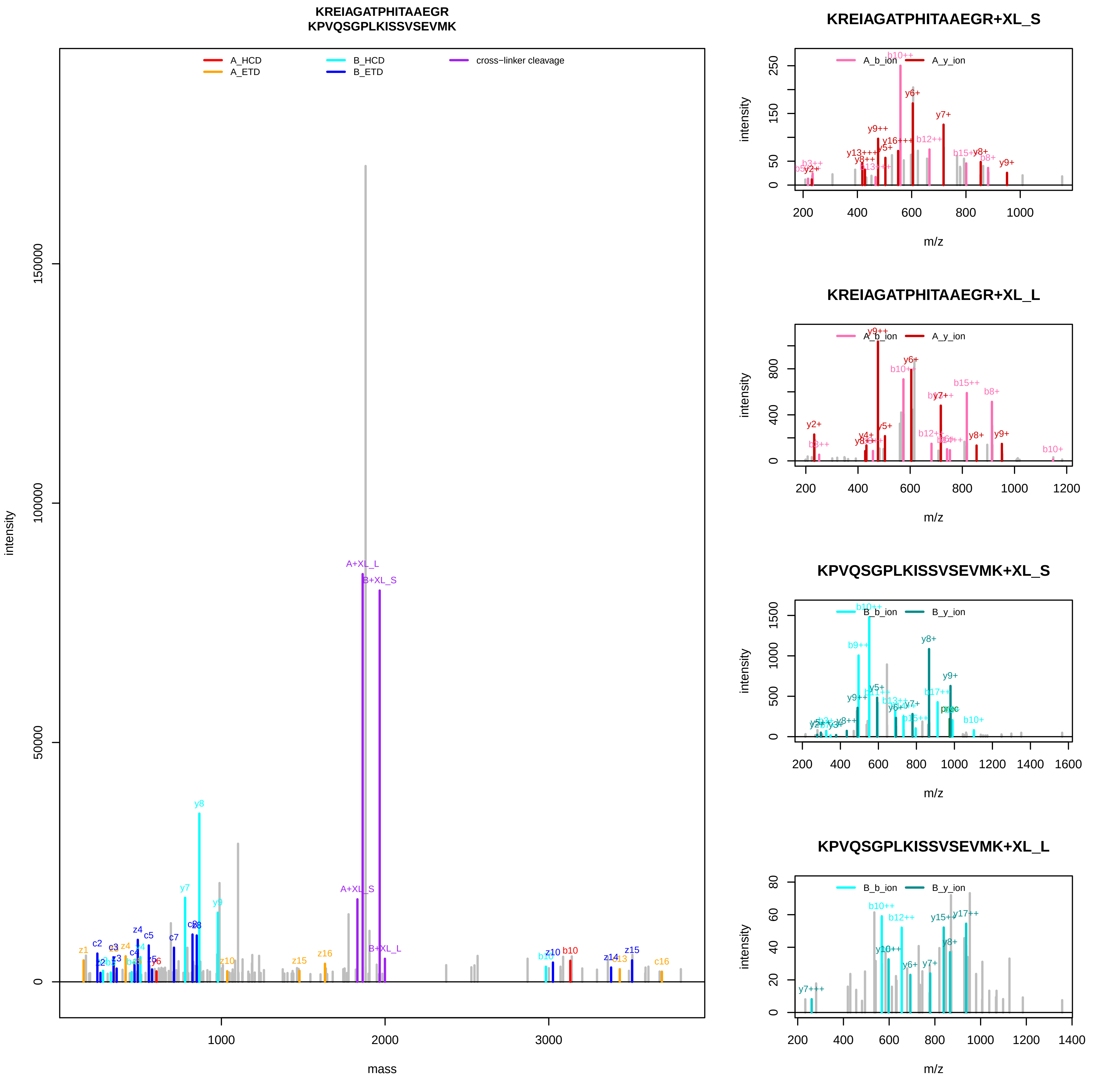
KREIAGATPHITAAEGR
KPVQSGPLKISSVSEVMK
A_HCD
A_ETD
B_HCD
B_ETD
cross−linker cleavage
A+XL_L
B+XL_S
A+XL_S
B+XL_L
y8
y7
y9
c2
c3
z3
z4
c5
c7
c8
z8
z10
z15
z16
b10
z14
z13
c16
intensity
mass
0
50000
100000
150000
1000
2000
3000
KREIAGATPHITAAEGR+XL_S
A_b_ion
A_y_ion
b10++
y6+
y7+
y9++
y16+++
b12++
y5+
y13+++
y8++
b15+
y8+
b8+
y9+
b3++
y2+
intensity
m/z
0
50
150
250
200
400
600
800
1000
KREIAGATPHITAAEGR+XL_L
A_b_ion
A_y_ion
y9++
y6+
b10++
b15++
b8+
y7+
y5+
y2+
y4+
y8+
y9+
b12++
b10+
intensity
m/z
0
400
800
200
400
600
800
1000
1200
KPVQSGPLKISSVSEVMK+XL_S
B_b_ion
B_y_ion
b10++
b9++
y8+
y9+
y5+
y9++
b17++
b13++
y6+
y7+
prec
b15++
b10+
y8++
intensity
m/z
0
500
1000
1500
200
400
600
800
1000
1200
1400
1600
KPVQSGPLKISSVSEVMK+XL_L
B_b_ion
B_y_ion
b10++
b12++
y15++
y17++
y10++
y8+
y6+
y7+
y7+++
intensity
m/z
0
20
40
60
80
200
400
600
800
1000
1200
1400
